# Small cell lung cancer transformation after EGFR-TKIs treatment in lung adenocarcinoma: A case report and literatures review

**DOI:** 10.1097/MD.0000000000032697

**Published:** 2023-01-27

**Authors:** Xiaoli Chen, Dongmei Li, Kun Miao, Tao Shou, Wenjing Zhang

**Affiliations:** a Medical School, Kunming University of Science and Technology, The First People’s Hospital of Yunnan Province, Kunming, China; b Department of Medical Oncology, The First People’s Hospital of Yunnan Province, Kunming, China.

**Keywords:** adenocarcinoma, epidermal growth factor receptor (EGFR), small cell transformation, T790M mutation

## Abstract

Epidermal growth factor receptor-tyrosine kinase inhibitors (EGFR-TKIs) are commonly used in the treatment of advanced non-small cell lung cancer. However, patients will inevitably develop resistance to EGFR-TKIs in the long-term treatment process. In this paper, we report a case of small cell lung cancer transformation after EGFR-TKIs treatment in lung adenocarcinoma. We summarize the characteristics of this case and the treatment after transformation, and emphasized the repeat biopsy and dynamic monitoring its genetic mutation was necessary.

**Patient concerns:**

A 75-years-old man with no smoking history was admitted to our hospital with repeated cough and expectoration for 1 month and chest enhancement computed tomography showed paracbronchial soft tissue mass in the lower lobe of the left lung, which was considered to be central lung cancer.

**Diagnoses:**

The first pathological analysis of lung biopsy confirmed left lung adenocarcinoma and clinical stage was T3N3M1 IVA. In June 2021, the second bronchoscopic biopsy was performed, and pathology showed small cell neuroendocrine carcinoma in the left lung.

**Interventions:**

Gefitinib was given to patients when the first next generation sequence test showed EGFR L858 mutation. When the second next generation sequence test revealed EGFR T790M mutation, the patient received with osimertinib. The patient got 2 cycles chemotherapy of etoposide plus netaplatin when diagnosed with small cell lung cancer.

**Outcomes:**

Progression-free survival was only 8 months after gefitinib treatment. Moreover, the patient was insensitive to Oxitinib, and the disease progressed after 2 months of treatment with Oxitinib. Finally, he died of severe infection and hepatic failure after a diagnosis of small cell lung cancer.

**Lessons:**

Our case highlights that if a patient has rapid disease progression, increase of serum neuron-specific enolase, and TP53 and Rb1 inactivation during EGFR-TKIs treatment, we should be alert to the pathological type transformation to small cell lung cancer.

## 1. Introduction

Gene detection is a conventional treatment strategy for advanced lung adenocarcinoma, and common mutation types include epidermal growth factor receptor (EGFR), anaplastic lymphoma kinase mutation and C-ros oncogene 1-receptor tyrosine kinase (ROS1) fusion.^[1]^ The common mutation types of EGFR are exon 19 deletion (19DEL) and exon 21 L858R point mutation (21L858R).^[2]^ EGFR has tyrosine kinase activity. It plays an important role in the occurrence and development of cancer. For the above mutations, molecular targeting, namely EGFR-Tyrosine kinase inhibitors (EGFR-TKIs), can be used for treatment. Relevant studies have confirmed that the use of this treatment can significantly improve the quality of life of patients, prolong the survival period, and bring significant benefits to patients.^[3,4]^ However, drug resistance is inevitable in the long course of treatment, and the occurrence of small cell transformation is one of the important mechanisms of drug resistance. Here, we report a case of small cell lung cancer transformation after EGFR-TKIs treatment in lung adenocarcinoma, and discuss its characteristics and related mechanisms.

## 2. Case presentation

**Figure 1.**
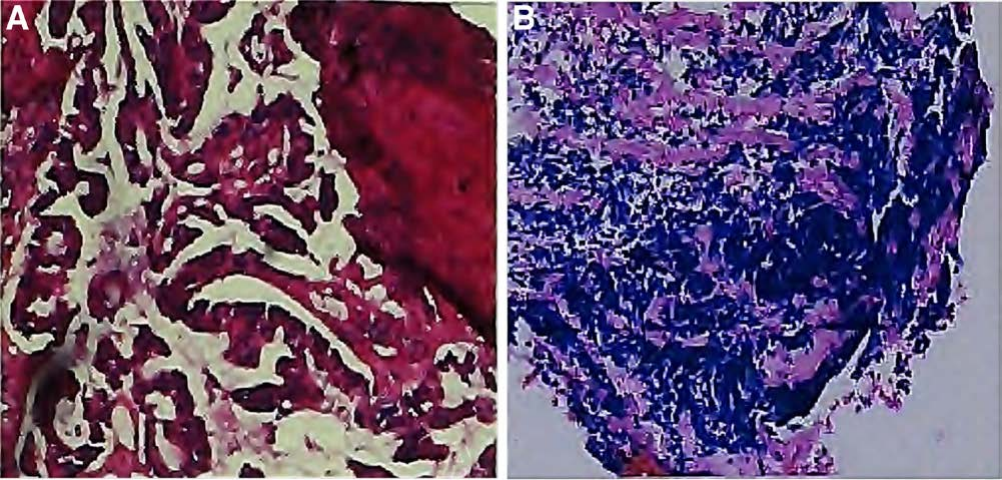
Hematoxylin and Eosin staining of left lung biopsy showed adenocarcinoma on July 2021 (A), and Hematoxylin and Eosin staining of left lung tissue revealed small cell neuroendocrine carcinoma on July 2021 (B).

A 75-years-old man with no smoking history was admitted to our hospital with repeated cough and expectoration for 1 month and chest enhancement computed tomography (CT) showed paracbronchial soft tissue mass in the lower lobe of the left lung, which was considered to be central lung cancer. Lung biopsy pathology showed adenocarcinoma of the left lung (cT3N3M1 IVA) on July 2020 (Fig. 1A). He achieved 1 cycle of chemotherapy with pemetrexed plus bevacizumab, without platinum regimen due to the Eastern Cooperative Oncology Group performance status (ECOG PS) 2. Two weeks later, the result of next generation sequence (NGS) with biopsy tissue showed that the patient harboring an EGFR L858 mutation. He obtained a clinically significant response for about 8 months through the treatment of gefitinib.

After progression, the second NGS test showed that EGFR L858R T790M mutation and PIK3CA E545K mutation. Oxitinib was then administrated. However, he presented abdomen pain only 2 months later and CEA (254.38 ng/mL) was lower than before, while neuro-specific enolase (NSE) (27.32 ng/mL) was higher than before, pathological bronchoscopy of the left lung revealed small cell neuroendocrine carcinoma (Fig. 1B), with EGFR L858R mutation, RICTOR copy number increased and PD-L1 < 1% on July 2021. The patient received 2 cycles chemotherapy of etoposide plus netaplatin, and got partial response. CEA (209.57 ng/mL) and NSE (15.25 ng/mL) were both lower than before. However, he refused to continuous chemotherapy due to the side effects. The patient was then treated with Icotinib and Anlotinib.

**Figure 2.**
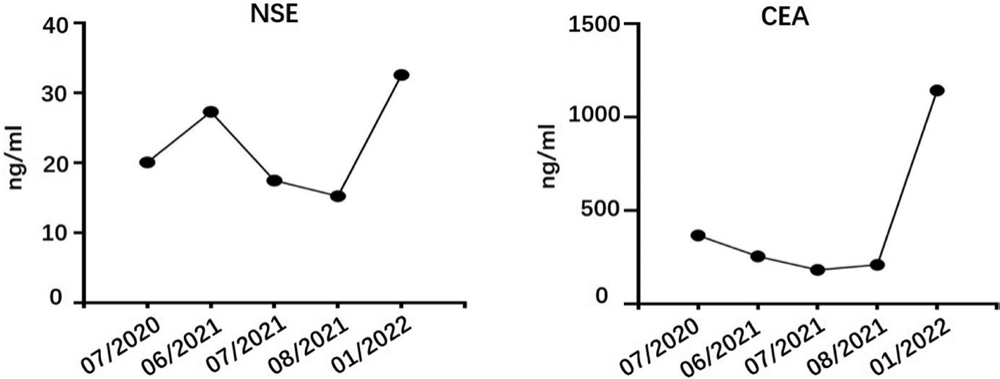
The change of NSE and CEA level during the treatment. CEA = carcinoembryonic antigen, NSE = neuro-specific enolase.

**Figure 3.**
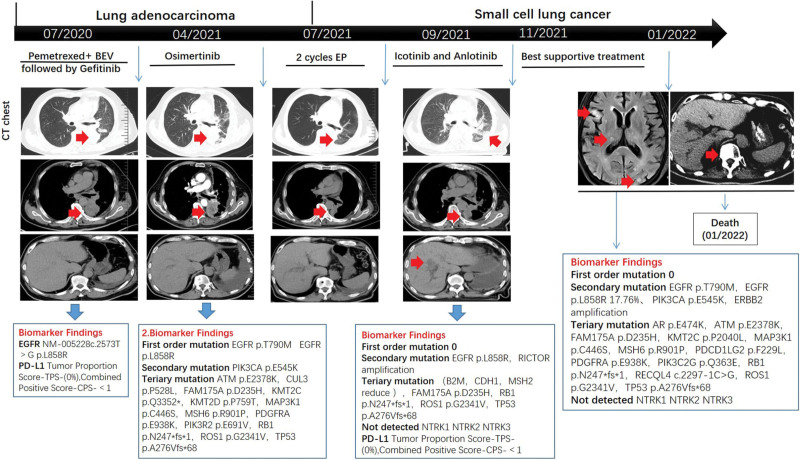
Case presentation of transformation of lung adenocarcinoma to small cell lung carcinoma after EGFR-TKI treatment. CT = computed tomography, EP = etoposide plus netaplatin, HE = hematoxylin and eosin.

After 5 months, the disease progressed extensively with bone, liver and multiple lymph node metastases. Whole blood NGS test in January 20, 2022 showed EGFR L858R and T790M mutation, PIK3CA e545K mutation, and the copy number of ERBB2 increased. CEA (1142.66 ng/mL) was 5-times higher than that before and NSE (32.59 ng/mL) was 1-time higher than that before (Fig. 2). According to the genetic test results, the patient was treated with oxitinib. Unfortunately, the patient passed away due to severe infection and hepatic failure on January 23, 2022 on the third day after taking the drug (Fig. 3).

## 3. Discussion

The median progression free survival of advanced non-small cell lung cancer (NSCLC) patients with EGFR-sensitive mutations treated with first-generation EGFR-TKI is about 10 to 12 months.^[5–7]^ This patient had a shorter Progression-free survival after gefitinib treatment (8 months) than the reported data. More than surprise, he was T790M mutation-positive but had no benefit from third-generation EGFR-TKI. The reason for this is maybe because he has several co-mutated genes or because of the heterogeneity of tumors, different subclones are produced between tumor tissues, which leads to the sensitivity and specificity of whole blood NGS test not reaching 100%, so patients cannot benefit from third-generation EGFR-TKI.^[8]^Marcoux et al studied the tissue genotyping of 59 patients with translational small cell lung cancer (SCLC) with EGFR mutation and found that all patients retained the initial EGFR mutation, 15 of the 19 patients with positive EGFR T790M mutated into T790 wild type when converted to SCLC, and other mutations include TP53, Rb1 and PIK3CA, as well as the creation of other new mutations. Moreover, central nervous system metastasis often occurs after transformation to small cell lung cancer.^[9]^ This is consistent with the case in which the original EGFR mutation was retained, T790 became wild-type, and brain metastases occurred after transformation.

At present, there are 2 mechanisms of EGFR-TKIs resistance that have been further studied. First, EGFR-T790M mutation occurs during the treatment of EGFR-TKIs, which causes the loss of EGFR-TKIs activity and leads to drug resistance.^[10,11]^ Second, the amplification of MET receptor tyrosine kinases leads to the activation of EGFR-independent downstream intracellular signaling, resulting in drug resistance.^[12,13]^ In addition, there are still unclear mechanisms of resistance such as pathological type conversion (NSCLC to SCLC), KRAS, PIK3CA, TP53 and Rb1 mutations.

For the transformational small-cell lung cancer, there is no definite standard treatment guidelines, based on the primary small cell lung cancer treatment strategy, still USES platinum-based double medicine treatment.^[14]^ In this case, EP protocol was used after transformation. After review, the lesion was smaller than before, and the patient was sensitive to this treatment. However, it has the same biological characteristics as the original SCLC, easy to relapse, rapid disease progression, and easy to occur central nervous system metastasis, and the patient showed the above characteristics after transformation into small cell lung cancer.

## 4. Conclusions

At present, the mechanism of NSCLC transforming into SCLC after EGFR-TKIs treatment is still unclear, which may be one of the potential mechanisms of drug resistance. In the future clinical diagnosis and treatment, if the disease is found to be insensitive to EGFR-TKIs treatment, with rapid disease progression, rapid increase of CEA and NSE, and accompanied by TP53 and Rb1 inactivation, the transformation of pathological types should be vigilant. At this time, repeat biopsy is necessary. Moreover, dynamic detection of gene mutation and timely adjustment of treatment plan can bring the greatest benefit to patients.

## Acknowledgments

All of us thank the patient for consent to the publication of the case.

## Author contributions

**Formal analysis:** Kun Miao, Tao Shou.

**Investigation:** Kun Miao.

**Methodology:** Dongmei Li.

**Resources:** Tao Shou, Wenjing Zhang.

**Writing – original draft:** Xiaoli Chen.

**Writing – review & editing:** Wenjing Zhang.
